# POPISK: T-cell reactivity prediction using support vector machines and string kernels

**DOI:** 10.1186/1471-2105-12-446

**Published:** 2011-11-15

**Authors:** Chun-Wei Tung, Matthias Ziehm, Andreas Kämper, Oliver Kohlbacher, Shinn-Ying Ho

**Affiliations:** 1 School of Pharmacy, Kaohsiung Medical University, Kaohsiung 807, Taiwan; 2 Institute of Bioinformatics and Systems Biology, National Chiao Tung University, Hsinchu 300, Taiwan; 3 Center for Bioinformatics Tübingen, Eberhard Karls University Tübingen, 72076 Tübingen, Germany; 4 Department of Biological Science and Technology, National Chiao Tung University, Hsinchu 300, Taiwan

## Abstract

**Background:**

Accurate prediction of peptide immunogenicity and characterization of relation between peptide sequences and peptide immunogenicity will be greatly helpful for vaccine designs and understanding of the immune system. In contrast to the prediction of antigen processing and presentation pathway, the prediction of subsequent T-cell reactivity is a much harder topic. Previous studies of identifying T-cell receptor (TCR) recognition positions were based on small-scale analyses using only a few peptides and concluded different recognition positions such as positions 4, 6 and 8 of peptides with length 9. Large-scale analyses are necessary to better characterize the effect of peptide sequence variations on T-cell reactivity and design predictors of a peptide's T-cell reactivity (and thus immunogenicity). The identification and characterization of important positions influencing T-cell reactivity will provide insights into the underlying mechanism of immunogenicity.

**Results:**

This work establishes a large dataset by collecting immunogenicity data from three major immunology databases. In order to consider the effect of MHC restriction, peptides are classified by their associated MHC alleles. Subsequently, a computational method (named POPISK) using support vector machine with a weighted degree string kernel is proposed to predict T-cell reactivity and identify important recognition positions. POPISK yields a mean 10-fold cross-validation accuracy of 68% in predicting T-cell reactivity of HLA-A2-binding peptides. POPISK is capable of predicting immunogenicity with scores that can also correctly predict the change in T-cell reactivity related to point mutations in epitopes reported in previous studies using crystal structures. Thorough analyses of the prediction results identify the important positions 4, 6, 8 and 9, and yield insights into the molecular basis for TCR recognition. Finally, we relate this finding to physicochemical properties and structural features of the MHC-peptide-TCR interaction.

**Conclusions:**

A computational method POPISK is proposed to predict immunogenicity with scores which are useful for predicting immunogenicity changes made by single-residue modifications. The web server of POPISK is freely available at http://iclab.life.nctu.edu.tw/POPISK.

## Background

**Figure 1:**
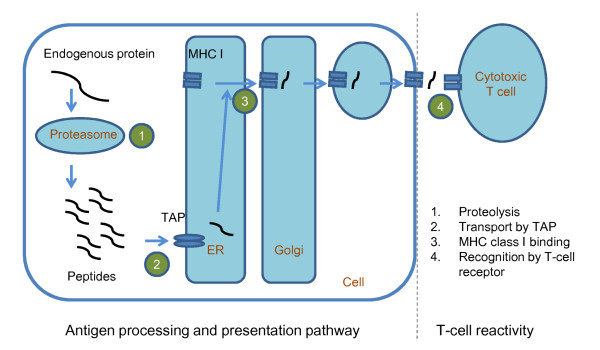
**The immunogenic pathway associated with MHC class I molecules**.

Immunogenicity is the ability to induce an immune response. For the major histocompatibility complex (MHC) class I-mediated immune response, this immune activation entails a successful processing of the antigen, its presentation by an MHC class I molecule and finally its recognition by a T-cell receptor (Figure 1). The predictions of antigen processing and MHC-peptide binding are well-studied problems in immunoinformatics. The prediction of T-cell reactivity, in contrast, is less well studied and much more difficult.

For computer-aided vaccine designs [1-3], the prediction of the immunogenicity is an important step. Computational methods for immunogenicity prediction accelerate the design of peptide-based vaccines. The immunogenic pathway can be split in two major phases as shown in Figure 1. Phase I includes all processes involving the antigen-presenting cell. For MHC class I, this phase encompasses proteasomal cleavage, peptide transport, the binding of a peptide to the MHC, and its presentation on the cell surface. Phase II is the recognition of this MHC-peptide complex by T cells leading to T-cell activation. Thus, a peptide has to fulfill at least two requirements to become immunogenic. First, the peptide has to be presented by an MHC molecule. Second, the T-cell receptor (TCR) has to bind to this peptide-MHC complex such that an immune response is triggered. Hence, overall immunogenicity is governed by antigen processing as well as MHC binding in Phase I, and mostly by T-cell reactivity in Phase II. For simplicity's sake, we summarily refer to Phase II, T-cell reactivity, as immunogenicity in the context of this work.

Numerous methods have been reported to predict individual steps of Phase I. We mention only selected works here and refer to recent reviews for a more complete picture [4-6]. There are several existing prediction methods for antigen cleavage [7-9], transport through the transporter associated with antigen processing (TAP) [10,11], and in particular for MHC-peptide binding. Techniques for predicting MHC binding include SYFPEITHI [12,13], BIMAS [14], SVMHC [15,16], NetMHC [17], NetMHCpan [18], KISS [19], RANKPEP [20,21], SVRMHC [22-24] and DynaPred [25]. These methods have typical prediction accuracies of almost 70-90%. Furthermore, there are techniques combining all three major steps of the antigen processing and presentation pathway [26-29].

It is commonly assumed that a peptide's immunogenicity is related to its binding affinity to MHC. However, recent studies demonstrated that the binding affinity to MHC class I molecules does not strongly correlate with the strength of induced T-cell immune responses [30-32]. Feltkamp et al. showed that the binding affinity to MHC class I molecules is required but does not ensure T-cell immune responses [33]. Furthermore, factors other than MHC binding affinity are found to strongly influence T-cell immune responses, compared with only moderate influence of MHC binding affinity [34]. All together, peptides predicted to be cleaved by proteasome and bound by TAP and MHC molecules have potential to be immunogenic but are not always immunogenic. The prediction and characterization of peptide immunogenicity will be valuable for better understanding the immune system.

In contrast with the numerous studies of dealing with antigen processing, only a few studies address Phase II by considering the T-cell immune responses involved. Prediction of immunogenicity is hard because it depends on the host immune system, in particular on the HLA and TCR types present in the immune repertoire. Besides common structural features of the MHC-peptide-TCR complex, immunogenicity is also governed by negative T-cell selection (central tolerance). In contrast with the influence of structural features, central tolerance as a property of the whole proteome cannot easily be learned. It is desirable to better characterize the peptide immunogenicity and develop methods for predicting immunogenicity of MHC-binding peptides.

In previous studies on the formation of the TCR-peptide-MHC complex, crystal structures have been analyzed [35-37] to correlate structural features of the TCR with immunogenicity and to identify TCR recognition positions. However, due to the small number of available crystal structures of the ternary complex, these are just case studies, with limited potential for generalization. For example, two studies found different important positions of HLA-A2 binding peptides for TCR recognition (position 8 [37] and positions 4 and 6 [35]). As an alternative approach to T-cell reactivity, experiments with substitutions and cytotoxicity assays have been performed for HLA-B27 [38]. However, so far results are based on only a few peptides. Furthermore, the relation between peptide sequence variation and immunogenicity that has not been convincingly demonstrated is important to better understand the immune system. Large-scale analyses are thus desirable to better characterize the relation between peptide sequences and immunogenicity, and the important positions of MHC binding peptides for immunogenicity.

The first predictor for T-cell reactivity published is POPI [31]. POPI is a support vector machine (SVM)-based method using 23 informative physicochemical properties of MHC class I binding peptides. While POPI performs reasonably well, it uses averaged values of physicochemical properties to represent peptides independent of the amino acid positions for T-cell reactivity. The method thus cannot yield structural insights into T-cell reactivity.

In this work, we investigate a systematic approach to prediction and analyses of T-cell reactivity by considering the effects of MHC restriction on immunogenicity. In order to better characterize the immunogenicity induced by MHC class I binding peptides and identify important positions of these peptides, we propose a prediction method (named POPISK) using SVM with string kernels that have been successfully applied in classification tasks [19,39-42]. This work establishes a large dataset IMMA2 by collecting immunogenicity data from three major immunology databases, MHCPEP [43], SYFPEITHI [12,13] and IEDB [44].

The method POPISK performs reasonably well in predicting peptide immunogenicity of HLA-A2 binding peptides where the mean 10-fold cross-validation accuracy is 0.68. For fair comparisons, a modified POPI method with physicochemical properties was implemented using the same dataset IMMA2. POPISK is better than the modified POPI with the accuracy of 0.60. In an analysis of seven HLA-A2-binding peptides with known crystal structures, POPISK accurately predicts the immunogenicity for the majority of peptides and successfully predicts the immunogenicity change of single-residue modifications reported in previous studies [45,46]. The results reveal that peptide sequence variation plays an important role in immunogenicity. We also analyzed importance of amino acid positions of the peptides with length 9 by selecting positions whose deletion significantly decreases prediction performance. The result shows that six positions (1, 4, 5, 6, 8 and 9) of HLA-A2 binding peptides are important for T-cell reactivity and thus immunogenicity. As a confirmation, graphical analyses using two sample logos [47] identified important positions 4, 6, 8 and 9. This finding is related to physicochemical properties and structural features of the MHC-peptide-TCR interaction.

## Methods

### Datasets

We establish a new and large dataset IMMA2 by extracting peptide binders of length 9 with associated human MHC class I alleles and their corresponding immunogenicity data from the three databases MHCPEP [43], SYFPEITHI [12,13] and IEDB [44]. Although the MHCPEP database has not been updated since 1998, it is still widely used for analysis [48-51]. By using three different databases, the experimental results are expected to have no bias towards any one of the data sources.

For the MHCPEP database, the peptide sequences and their associated MHC alleles, binding and immunogenicity data are extracted from the fields of 'SEQUENCE', 'MHC MOLECULE', 'BINDING' and 'ACTIVITY', respectively. The 'BINDING' field annotates a peptide as either a binder or a non-binder. There are four levels (none, little, moderate and high) of immunogenicity in MHCPEP that can be obtained from the field "ACTIVITY". Peptides annotated as 'none' in the field "ACTIVITY are non-immunogenic peptides. Peptides annotated as the other three levels are immunogenic peptides.

For the IEDB database, the peptide sequences and their associated MHC alleles, qualitative binding and qualitative immunogenicity data are extracted from the fields of 'Epitope', 'MHC Restriction', 'MHC binding', and 'T cell response', respectively. Only peptides with positive binding annotation were selected for analyses. A peptide with positive annotation in the field of 'T cell response' is an immunogenic peptide. In contrast, a peptide with negative annotation in the field of 'T cell response' is a non-immunogenic peptide. Unlike the databases MHCPEP and IEDB, there are only immunogenic peptides in the SYFPEITHI database. For the SYFPEITHI database, immunogenic peptides associated with various MHC alleles are extracted from the field of 'T-Cell epitopes'.

These peptide sequences were grouped into allele-specific datasets according to their associated HLA supertypes [52]. In order to utilize all available data for analyzing immunogenicity conferred by any of TCRs, peptides with contradictory annotations (immunogenic and non-immunogenic) were regarded as immunogenic peptides. That means a peptide recognize by any of TCRs is an immunogenic peptide. Similarly, the identified sequence patterns would be recognized by any of TCRs. Despite thousands of extracted entries are available for many alleles, there is only one allele HLA-A2 with enough data (> 500 peptides) for subsequent analysis after removing duplicate entries. The main reason for high duplication rate is the use of different methods and conditions for measurement of immunogenicity. Therefore, this study focuses on HLA-A2, one of the best known allele with plenty of previous knowledge for comparison with findings from this study. Also, due to the small number of peptides associated with the other alleles, it is hard to create robust models for the other alleles. The dataset of allele HLA-A2 (named IMMA2) consists of 558 immunogenic and 527 non-immunogenic peptides and is available at http://iclab.life.nctu.edu.tw/POPISK/download.php.

### The proposed method POPISK

POPISK (prediction of peptide immunogenicity using string kernel) uses support vector machines (SVMs) with a weighted degree string kernel. SVMs cope well with the over-fitting problem arising from a small training dataset by finding a linear separation hyperplane that maximizes the distance between two classes to create a classifier. SVMs can efficiently deal with classification, prediction and regression problems. Given training vectors **x***_i _*∈ R*^n ^*and their class values *y_i _*∈ {-1, 1}, *i *= 1, ..., *N*, an SVM solves the problem of minimizing $\frac{1}{2}w^{\text{T}}w+C\overset{N}{\underset{i=1}{\sum }}\xi_{i}$, subject to *y_i_*(**w**^T^**x***_i_*+*b*) ≥ 1-*ξ_i _*and *ξ_i _*≥ 0, where **w **is a normal vector perpendicular to the hyperplane and *ξ_i _*are slack variables allowing for some misclassifications. The cost parameter *C *> 0 controls the trade-off between the margin and the training error. Larger values of *C *will lead to a higher error penalty.

An effective weighted degree string kernel [41,53] counting the numbers of matched sub-sequences of length *p *at corresponding positions of two sequences is applied to transform samples to high-dimensional space to make linear separation easier. Given two sequences s*_i _*and s*_j _*of equal length *L *and degree *d*, the weighted degree string kernel computes the total numbers of matched sub-sequences of length *p *∈ {1, ..., *d*} at corresponding positions *l *of two sequences, defined as follows:

(1) $$k\left({\text{s}}_{i},{\text{s}}_{j}\right)=\overset{d}{\underset{p=1}{\sum }}\beta_{p}\overset{L-p+1}{\underset{l=1}{\sum }}I\left(u_{p,l}\left({\text{s}}_{i}\right)=u_{p,l}\left({\text{s}}_{j}\right)\right),$$

where *I(h) = *1 if *h *is true; otherwise, *I(h) = *0, *u_p, l_*(s) is the sub-sequence of length *p *starting from position *l *of peptide sequence s, and *β_p _*are weighted coefficients. In this study, sequence length *L *is 9. The fixed values of *β_p _*= 2(*d*-*p*+1)/(*d*(*d*+1)) are adopted as used in the previous study [41]. Shogun [54,55] was used and LIBSVM [56] was chosen for implementation of the proposed method.

### Identifying informative physicochemical properties

Identification of informative physicochemical properties of peptides provides a better understanding of the TCR-peptide-MHC interaction. Since decision tree learning methods reveal interpretable rules, it is helpful to reveal differences between immunogenic and non-immunogenic peptides. We employed C5.0, a decision tree learning method, which is an improved version of C4.5 [57]. In C5.0, the information gain is utilized to rank features for constructing a decision tree by iteratively appending nodes with high ranks. After construction of a decision tree, C5.0 will automatically calculate feature usage for each feature by counting the firing frequency of associated rules (nodes). The feature usage provides an easy way to rank and identify important features. A physicochemical property with high feature usage is an important feature.

In this study, a total of 531 physicochemical properties without 'NA' values were retrieved from version 9.0 of the amino acid index (AAindex) database [58]. Each physicochemical property consists of a set of 20 numerical values for amino acids. The physicochemical properties have been extensively used for quantitative structure-activity relationship (QSAR) model [59-62] and for predicting MHC binding peptides [48,50,60,63,64]. To use physicochemical properties to represent a peptide, the peptide of *L *amino acids is encoded as an *L*-dimensional vector for each of the 531 physicochemical properties. The feature vector consisting of 531 mean values for representing physicochemical properties of the peptide can be obtained by separately averaging values of 531 *L*-dimensional vectors [31,65,66]. Please note that this study utilizes physicochemical properties only for analyzing their effects on TCR-peptide-MHC interactions. The proposed POPISK is based on SVM with string kernels using only sequence information.

### Comparison between POPISK and POPI

To the best of authors' knowledge, our recent work POPI [31] is the only method for predicting T-cell reactivity of MHC binding peptides. POPI is an SVM-based method using a radial basis function kernel and 23 informative physicochemical properties mined by using an inheritable bi-objective genetic algorithm. It is not fair to directly compare the results of POPISK with POPI because POPI is a four-class prediction method that predicts a peptide as highly, medium, little and not immunogenic. Furthermore, POPI is based on a smaller dataset. In order to perform a fair comparison, a modified POPI method (POPI-modified) was implemented using the dataset IMMA2 and the same 23 informative physicochemical properties for the binary prediction problem of immunogenic and non-immunogenic peptides.

### Performance evaluation

Three measurements are used to evaluate prediction performances of weighted degree kernel and SVM on the dataset IMMA2, namely overall accuracy (ACC), Matthew's correlation coefficient (MCC) and area under receiver operating characteristic curve (AUC):

(2) $$\text{ACC = }\frac{\text{TP + TN}}{\text{TP + TN + FP + FN}},$$

(3) $$\text{MCC = }\frac{\text{TP}\times \text{TN - FP}\times \text{FN}}{\sqrt{\left(\text{TP + FN}\right)\times \left(\text{TP + FP}\right)\times \left(\text{TN + FP}\right)\times \left(\text{TN + FN}\right)}},$$

where TP, TN, FP and FN are the number of true positives, true negatives, false positives and false negatives, respectively.

## Results and Discussion

The performance evaluation of POPISK is given as follows. First, we evaluate POPISK in predicting peptide immunogenicity. Second, we show the identification of important positions of a peptide for immunogenicity and give the differences found between immunogenic and non-immunogenic peptides. Third, physicochemical properties and their position-specific effects on immunogenicity are analysed. Finally, we present the prediction web server of POPISK and evaluate its performance.

### Prediction of peptide immunogenicity

To accurately predict immunogenicity of HLA-A2 binding peptides, it is necessary to tune two parameters (cost parameter *C *of the SVM and degree *d *of the weighted degree kernel) to build an accurate SVM classifier. In this study, a nested 10-fold cross-validation (10-CV) procedure was adopted to evaluate the prediction performance of our string kernel-based SVM classifier as it provides an almost unbiased estimate of the prediction error [67].

The nested 10-CV consists of two cross-validation loops: an inner loop for tuning SVM parameters and an outer loop for evaluating the test performance of the tuned SVM classifiers. First, the dataset IMMA2 was randomly divided into ten subsets of approximately equal size. For each iteration *m *(outer loop), the *m*-th subset is left out for testing the tuned SVM classifier trained by using the selected optimal parameter values giving the highest AUC performance using 10-CV on the remaining dataset (inner loop). The grid search method is applied to tune the parameters *C *_∈ _{2^-4^, 2^-3^, ..., 2^4^} and *d *∈ {1, 2, ..., 9}.

**Figure 2:**
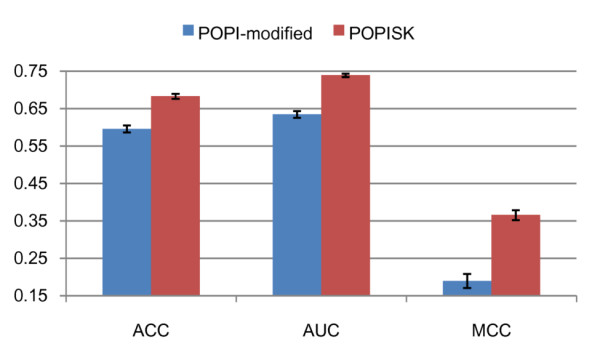
**Comparison of nested 10-CV performances of POPISK and POPI-modified**.

To obtain a robust statistical estimation of prediction performances, 20 independent runs of the nested 10-CV procedure were performed where the means and standard deviations of three performance measurements are regarded as final prediction performances. The best values of *C *and *d *having the highest AUC value on the inner 10-CV loop are always 1.0 and 9, respectively. The means and standard deviations of POPISK on the dataset IMMA2 are 0.68 and 0.007 for ACC, 0.74 and 0.004 for AUC, and 0.37 and 0.013 for MCC, respectively (Figure 2). The highest and lowest accuracies are 0.70 and 0.65 for ACC, 0.75 and 0.71 for AUC, and 0.30 and 0.40 for MCC, respectively. The small difference in prediction accuracies of 20 runs (200 predictions on randomly divided datasets) shows the robustness of the proposed method POPISK and the small effect of sequence similarities between training, validation and test datasets on the prediction performances. All nine string kernels and five complex string kernels provided by Shogun [54,55] were evaluated. Most of them perform similarly to or slightly worse than the weighted degree string kernel. Except for cost parameters *C *and degree parameter *d*, the above-mentioned results were obtained by using default values of parameters. All kernels might thus perform better by carefully tuning the respective parameters.

Previous studies for MHC binding predictions show that the use of quantitative data and regression methods is able to enhance the prediction performances [22-24]. However, currently there is only limited number (< 100) of HLA-A2 binding peptides with quantitative immunogenicity data in the databases of MHCPEP, IEDB and SYFPEITHI. The collection and utilization of quantitative immunogenicity data are expected to improve prediction performances and provide better functionality for immunologist.

### Comparison with POPI-modified

The evaluation procedure of the POPI-modified method is described as follows. First, the 23 informative physicochemical properties were used to encode peptides in the dataset IMMA2. Subsequently, 20 independent runs of the nested 10-CV were performed as follows. The grid search method was applied to tune the cost parameter *C *_∈ _{2^-4^, 2^-3^, ..., 2^4^} and the kernel parameter *γ *_∈ _{2^-4^, 2^-3^, ..., 2^4^} in the inner 10-CV loop. The SVM classifiers trained by using the selected parameters giving the highest AUC performance in inner 10-CV loop are used to evaluate the prediction performances in the outer 10-CV loop.

The comparison of nested 10-CV performances of POPISK and POPI-modified is shown in Figure 2. The nested 10-CV performances and corresponding standard deviations of POPI-modified are 0.60 and 0.009 for ACC, 0.64 and 0.009 for AUC and 0.19 and 0.018 for MCC, respectively. POPISK outperforms the POPI-modified method having 8% and 10% improvements for ACC and AUC, respectively.

**Figure 3:**
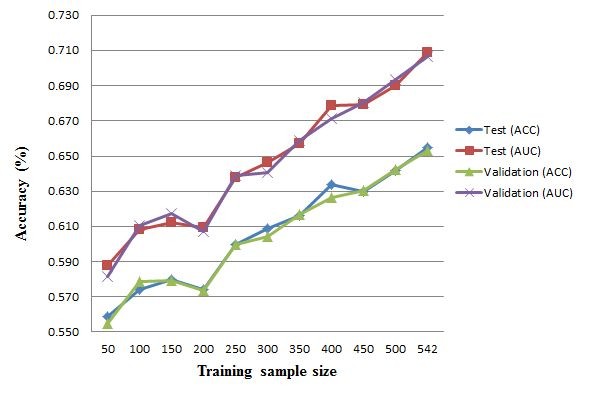
**Learning curves for various training sample sizes**.

To analyze the effect of sample size on the prediction performance of POPISK, a learning curve is designed to reveal the effect. First, the dataset IMMA2 is randomly divided into three dataset consisting of 50%, 25% and 25% peptides for training, validation and test datasets, respectively. For each training sample size *s *_∈ _{50, 100, 150, 200, 250, 300, 350, 400, 450, 500, 542}, the set of *s *randomly-selected samples is applied to train a SVM model. Subsequently, the validation and test datasets are used to evaluate the built model. Figure 3 shows the learning curves for various training sample sizes. The prediction accuracies of ACC and AUC for validation and test datasets increase as the training sample size increases. By collecting more data, POPISK is expected to perform better and can be applied to analyze immunogenicity of peptides associated with other MHC alleles.

### Identification of important positions for immunogenicity

Compared with the familiarity of MHC binding peptide's motifs, the understanding of T-cell recognition positions of MHC binding peptides is still not satisfactory. Some studies have aimed to identify the T-cell recognition positions. However, these studies were based on only a few crystal structures and identified different recognition positions [35-37]. The identification of important positions for immunogenicity will shed light on the mechanism of T-cell recognition and accelerate the development of peptide-based vaccines. To assess the individual contributions of each position of MHC-binding peptides to the prediction performance, we proposed two efficient methods to estimate the importance of positions, described as follows.

**Figure 4:**
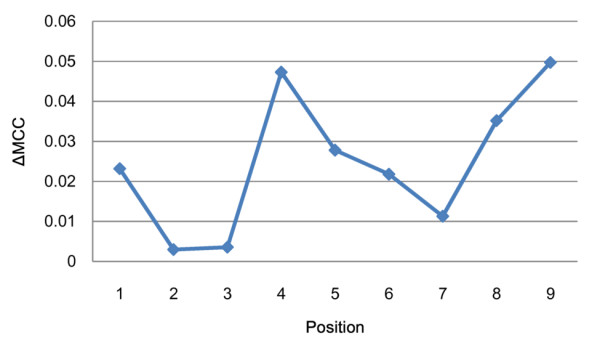
**The decrease in MCC performances evaluated on datasets without using residues in specific positions**.

The proposed method uses the decrease in prediction performance resulted from removing the amino acid on a specific position of the peptide to designate the importance for each position. The larger the decrease in performance, the greater the importance of the position is. The change in prediction performance is evaluated as follows. First, nine additional datasets for nine positions were created by removing amino acids in the corresponding positions from the dataset IMMA2. Subsequently, for each of the nine datasets, 20 runs of nested 10-CV were performed as described above to evaluate prediction performances. For the parameter tuning process, the largest value of degree parameter *d *is set to 8 (the same as the remaining peptide length). The decreases in performance as measured by MCC (ΔMCC) for these datasets are shown in Figure 4. Other performance measures (AUC, ACC) yield similar results (data not shown). Six positions (1, 4, 5, 6, 8 and 9) are identified as important positions since the prediction performance of those positions decreased significantly.

**Figure 5:**
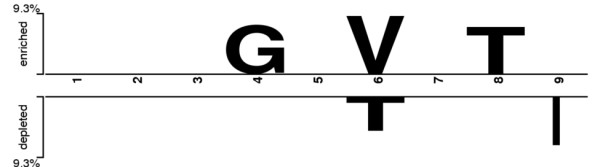
**Two-Sample Logo representation of over- (upper half) and underrepresented (lower half) residues in immunogenic peptides**.

To further investigate over- and underrepresented amino acids in corresponding positions, the two-sample logos [47] are computed to graphically represent the differences between immunogenic and non-immunogenic peptides of all peptides in IMMA2. The identified over- and under-represented amino acids in specific positions show the sequence preferences for recognitions by any of TCRs. Statistically significant residues selected by using a two-sample *t*-test with *p *< 0.05 are represented in the logo. In addition, a widely used multiple-comparison correction (Bonferroni correction) is applied to eliminate false positives by adjusting the significance level. Figure 5 shows the resulting two-sample logo representations. The residues overrepresented in immunogenic peptides (shown in the upper half of Figure 5) are glycine, valine and threonine at positions 4, 6 and 8, respectively. On the other hand, the residues underrepresented in immunogenic peptides (shown in the lower half of Figure 5) are threonine and isoleucine at positions 6 and 9, respectively.

Our method successfully identified previously reported TCR recognition positions (4, 6 and 8) for HLA-A2 binding peptides from an analysis of crystal structures [35-37]. Notably, the underrepresented residue isoleucine in position 9 is the anchor residue for peptides binding to HLA-A2 molecules [68]. However, position 2, the primary anchor position of HLA-A2 binding peptides [68,69], is not important for predicting peptide immunogenicity from a set of MHC-binding peptides.

The above findings might explain the observation that peptides with high binding affinity to MHC class I molecules do not always induce immune responses [30-34]. Because there are only 11 peptides without natural source in IMMA2, the identified sequence patterns are less likely derived from proteasome cleavage, TAP binding and MHC binding. Both analyses are based on only sequences. The use of feature-independent methods can avoid the bias derived from applied features. It is noteworthy that the average predicted affinity of non-immunogenic peptides is significantly stronger than that of immunogenic peptides (p < 0.05, *t*-test) in IMMA2. The results confirm the idea that although MHC binding is a prerequisite for immunogenicity but the peptide immunogenicity does not solely depend on binding affinity [30-34].

### Identification of informative physicochemical properties

Physicochemical properties play an important role in biomolecular recognition. The identification of informative physicochemical properties will provide insights into the underlying mechanism of immunogenicity. To identify the informative position-independent physicochemical properties, all HLA-A2 binding peptides were encoded as feature vectors with 531 mean values of physicochemical properties. Subsequently, C5.0 was applied to build a decision tree using the whole dataset IMMA2. The feature usage obtained from C5.0 can be used to rank the physicochemical properties. Table 1 shows physicochemical properties with usage larger than 50%.

**Table 1:** Physicochemical properties with feature usage larger than 50%

Usage	AAindex ID	Physicochemical properties
100%	MEEJ800102	Retention coefficient in HPLC, pH2.1
91%	WOLS870102	Principal property value z2
87%	CASG920101	Hydrophobicity scale from native proteins
84%	NAKH900110	Normalized composition of membrane proteins
81%	FASG760105	pK-C
79%	FAUJ880105	STERIMOL minimum width of the side chain
76%	CHAM830107	A parameter of charge transfer capability
61%	QIAN880127	Weights for coil at the window position of -6
59%	RACS820108	Average relative fractional occurrence in AR (i-1)
58%	DIGM050101	Hydrostatic pressure asymmetry index, PAI
56%	TANS770109	Normalized frequency of coil

Hydrophobicity (AAindex IDs MEEJ800102, CASG920101, NAKH900110 and FASG760105) is obviously a major contributor to predict peptide immunogenicity. Another property with AAindex ID WOLS870102 is correlated with molecular weight and residue volume, and probably relates to the limited space between MHC and TCR. Three properties (QIAN880127, RACS820108 and TANS770109) are related to secondary structure propensities and most likely indicate structural preferences of the peptide backbone.

**Figure 6:**
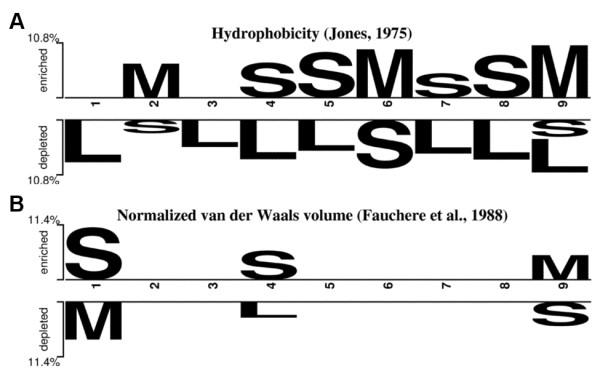
**The over- (upper half) and underrepresented (lower half) position-specific properties in immunogenic peptides**. (A) Hydrophobicity. (B) Normalized van der Waals volume. The symbols S, M and L indicate residues with small, medium and large hydrophobicity/volume, respectively.

To further investigate the position-dependent effect of informative physicochemical properties, two properties were selected to encode amino acids of IMMA2 peptides to two three-alphabet sequences (small (S), medium (M) and large (L)): hydrophobicity (thresholds 0.5 and 2.5) [70] and normalized van der Waals volume (thresholds 2.0 and 6.0) [71]. The encoded sequences yielded the two-sample logos shown in Figure 6. Both primary and secondary anchor positions for MHC binding (positions 2 and 9, respectively) and position 6 prefer residues of medium hydrophobicity (Figure 6A). Positions 4, 5, 7 and 8 prefer residues of small hydrophobicity. Positions 1 and 4 prefer residues with small van der Waals volume (Figure 6B) whereas position 9 prefers medium volume residues. The logos obtained by using the other volume-related properties are similar to Figure 6B.

### Web server of POPISK

The web server of POPISK was implemented by training an SVM classifier using the weighted degree string kernel (parameters *C *= 1.0 and *d *= 9) on the whole dataset IMMA2. Users can either input a peptide sequence of length 9 that binds to HLA-A2 molecules or upload a file of multiple 9-mer sequences. POPISK will output the predicted immunogenicity (immunogenic or non-immunogenic) accompanied with a score (decision value of SVM) for the strength of immunogenicity. Peptides with a decision value larger than zero are considered immunogenic. The web server of POPISK is publicly available at http://iclab.life.nctu.edu.tw/POPISK.

### Prediction and analysis using crystal structures

To further evaluate the prediction and analysis abilities of POPISK, a total of 17 crystal structures consisting of TCRs, peptides of length 9, and HLA-A2 molecules were extracted from the Protein Data Bank (PDB) [72]. By removing entries with duplicate peptide sequences or modified amino acids, seven crystal structures (PDB ID: 1qrn, 1qse, 1qsf, 1ao7, 1oga, 2bnr and 2bnq) are used for the following analyses. These peptides are classified as immunogenic (1qse, 1ao7, 1oga, 2bnr and 2bnq) or non-immunogenic (1qrn and 1qsf) according to the original publications [37,45,46].

First, POPISK was trained by using a modified dataset that excludes peptides of the seven test peptides from IMMA2. Subsequently, POPISK was applied to predict the seven peptides. The prediction results are shown in Table 2. POPISK classified 5 out of 7 peptides correctly. Although the peptide of 1ao7 is misclassified, its score (-0.04) is very close to the decision threshold value, zero.

**Table 2:** Prediction results of POPISK

PDB ID	Sequence	Source	POPISK Score	Experimental immunogenicity*
1qrn	LLFGYAVYV	Modified Tax protein of HTLV-1	-0.26	-
1qse	LLFGYPRYV	Modified Tax protein of HTLV-1	-0.14	+
1qsf	LLFGYPVAV	Modified Tax protein of HTLV-1	-0.07	-
1ao7	LLFGYPVYV	Tax protein of HTLV-1	-0.04	+
1oga	GILGFVFTL	Matrix protein of influenza	1.10	+
2f53	SLLMWITQC	Cancer/testis antigen 1B	1.11	+
2bnq	SLLMWITQV	Modified Cancer/testis antigen 1B	1.36	+

*+: immunogenic peptide; -: non-immunogenic peptide

The scores predicted by POPISK are useful for predicting the immunogenicity change made by single-residue modifications. For example, the prediction results show that modified cancer/testis antigen with valine in position 9 (POPISK score: 1.36) is more immunogenic than the original antigen (POPISK score: 1.11) and are consistent with a previous study [45]. Also, compared with the original Tax protein of human T-lymphotropic virus (POPISK score: -0.04), the reduced immunogenicity of three modified Tax proteins (POPISK scores: -0.07, -0.14 and -0.26) as shown in a previous study [46] is successfully predicted.

**Figure 7:**
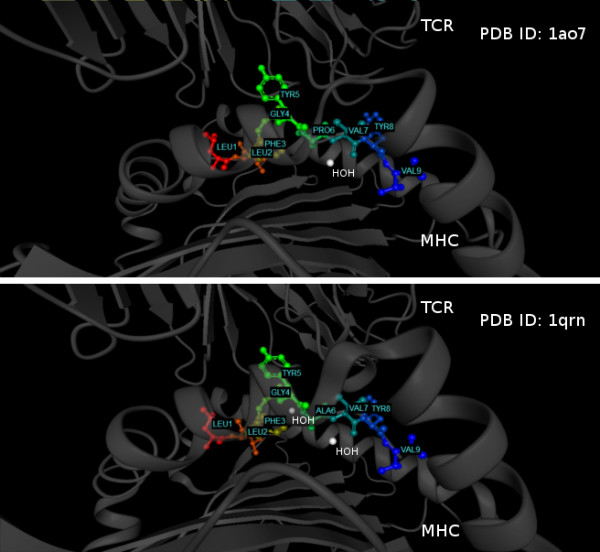
**Structures of PDB IDs **1ao7**and **1qrn. Structures of PDB IDs 1ao7 and 1qrn share high structural similarity presenting complexes of TCR-peptide-MHC. There is only one significant difference of the enlarged cavity at position 6 of the non-immunogenic peptide LLFGYAVYV in the 1qrn complex, compared with the immunogenic peptide LLFGYPVYV in the 1ao7 complex. Figure generated by BALLView 1.3 [74,75].

Among the seven TCR-peptide-MHC structures taken for our analyses, three different TCRs, the A6 TCR (1qrn, 1qse, 1qsf, 1ao7), the V_β_17V_α_10.2 TCR from the T-cell clone JM22 (1oga), and the 1G4 TCR (2bnr, 2bnq) are present. Hence, a comparison from the structural perspective can only be performed for each type of TCR individually. The most interesting peptide here is the A6 TCR, where structures with immunogenic as well as non-immunogenic peptides are available. The very high structural similarity among the structures of the A6 TCR has been stressed by Ding *et al*. [46]. These authors did not see any correlation between the overall shape of the complexes or rearrangements at the interface and immunogenicity. The highest overall structural similarity of complexes was found between the immunogenic peptide LLFGYPVYV (wild-type, 1ao7) with a POPISK score of -0.04 and the non-immunogenic peptide LLFGYAVYV (P6A, 1qrn) with a POPISK score of -0.26. Also, between the two peptides no difference in their solvent-accessible surface areas could be found. Figure 7 shows the two crystal structures of 1ao7 and 1qrn.

A significant difference between the two structures is the formation of an enlarged cavity at position 6 of the peptide in the P6A complex. An ordered water molecule entered this cavity, leading to some rearrangements of amino acids to accommodate the water. However, the formation of a cavity, the small rearrangements, and the entropic loss due to the conserved water account for only a fraction of the difference in complex dissociation constants [46]. A second difference was evident from shape complementarity analyses, showing a hole in the interface of P6A and a decrease in complementarity [73] affecting binding to residue at position 5.

For the modified cancer/testis antigen with valine in position 9, there is a subtle increase in the shape complementarity and the buried surface within MHC binding pocket compared with the original cancer/testis antigen with cysteine in position 9. The structural difference in the peptide is transmitted to the TCR and makes the TCR a slightly tilt [45]. Possible explanation of higher immunogenicity for the modified cancer/testis antigen might be the slightly better overall shape complementarity between TCR-peptide-MHC caused by a larger volume occupied by side chain of valine [45]. These findings show that even an in-depth structural analysis of the ternary complexes can only give hints on the immunogenicity of peptides, stressing the importance of large-scale statistical studies.

## Conclusions

The immunogenicity of peptides affected by intrinsic physicochemical properties and the extrinsic immunoglobulin repertoire determines the effectiveness of peptide vaccines and therapeutic peptides. Characterization of relation between peptide sequences and immunogenicity, and prediction of peptide immunogenicity will be valuable to the development of peptide vaccines. This study proposes a computational method POPISK based on support vector machines with a weighted string kernel to predict peptide immunogenicity and identify important recognition positions.

Compared with the only published predictor of T-cell reactivity, POPI [31], POPISK is more accurate (0.68 vs. 0.60) and yields insights into the relevance of specific sequence positions of the peptide for immunogenicity. A total of three central positions (4, 5 and 6) and three terminal positions (1, 8 and 9) of HLA-A2 binding peptides are identified as important positions for immunogenicity. Positions 4, 6 and 8 are separately identified by previous studies (position 8 [37] and positions 4 and 6 [35]). The two-sample logo method [47] confirms the important positions 4, 6, 8 and 9.

Physicochemical properties of peptides play important roles in determining immunogenic strength. In eleven informative properties selected by the decision tree method C5.0, four properties are hydrophobicity-related and two properties are residue volume-related. Compared with the structural analysis of ternary complexes, the good performance of the sequence-based prediction method POPISK implies that peptide sequence variations may play an important role in determining immunogenicity. Furthermore, POPISK successfully predicts the immunogenicity changes made by single-residue modifications. By collecting more data, POPISK is expected to perform better and can be applied to analyze immunogenicity of peptides associated with the other MHC alleles. The collection and utilization of quantitative immunogenicity data are expected to improve prediction performances as previous works for MHC binding predictions [22-24]. Finally, a freely available web server of POPISK for predicting peptide immunogenicity is established.

## Competing interests

The authors declare that they have no competing interests.

## Authors' contributions

CWT and OK conceived and designed the experiments. CWT and MZ implemented programs and performed the experiments. CWT, AK, OK and SYH analyzed the data. CWT, MZ, AK, OK and SYH wrote the paper. All authors read and approved the final manuscript.
